# Development and Application of an Immunoaffinity Column Enzyme Immunoassay for Mycotoxin Zearalenone in Complicated Samples

**DOI:** 10.1371/journal.pone.0085606

**Published:** 2014-01-17

**Authors:** Xiaoqian Tang, Xin Li, Peiwu Li, Qi Zhang, Ran Li, Wen Zhang, Xiaoxia Ding, Jiawen Lei, Zhaowei Zhang

**Affiliations:** 1 Oil Crops Research Institute of the Chinese Academy of Agricultural Sciences, Wuhan, P. R. China; 2 Key Laboratory of Biology and Genetic Improvement of Oil Crops, Ministry of Agriculture, Wuhan, P. R. China; 3 Key laboratory of Detection for Mycotoxins, Ministry of Agriculture, Wuhan, P. R. China; 4 Laboratory of Risk Assessment for Oilseeds Products (Wuhan), Ministry of Agriculture, Wuhan, P. R. China; 5 Quality Inspection and Test Center for Oilseeds Products, Ministry of Agriculture, Wuhan, P. R. China; University of California, Merced, United States of America

## Abstract

The zearalenone (ZEA) monoclonal antibody (mAb) 2D3, one of the highest sensitivity antibodies, was developed. Based on this mAb, it was established of an immunoaffinity column (IAC) coupled with an indirect competitive enzyme-linked immunosorbent assay (icELISA). After optimization, the icELISA allowed an IC_50_ against ZEA of 0.02 µg L^−1^. The mAb 2D3 exhibited a high recognition of ZEA (100%) and *β*-zearalenol (*β*-ZOL, 88.2%). Its cross-reactivity with *α*-zearalenol (*α*-ZOL) and *β*-zearalanol (*β*-ZAL) were found to be 4.4% and 4.6%, respectively. The IAC-icELISA method was employed to analyze ZEA contamination in food samples, compared with high-performance liquid chromatography (HPLC). The spiked assay for ZEA demonstrated the considerable recoveries for IAC-icELISA (83–93%) and HPLC (94–108%) methods. Results showed that the mAb 2D3 and IAC-icELISA method posed potential applications in sensitively determination of ZEA in maize.

## Introduction

Zearalenone (ZEA) is one common mycotoxin produced by several *Fusarium* species [1]. ZEA is a resorcylic acid lactone, which usually involved hyperestrogenism and other breeding disorders in pigs, sheep and other farm animals [2]. It is acknowledged that ZEA is relatively low in acute and chronic toxicity, carcinogenicity, and genotoxicity, and that it can induce DNA-adduct formation [3]. Meanwhile, ZEA can be widely found in cereals during cereal storage. Nowadays, tremendous evidences showed the global ZEA contamination in maize, barley, oats, wheat, rice and broomcorn [4].

Alhough ZEA is frequently found in maize, maize is still extensively used as a raw material in feeds or as essential food. Many countries and districts, such as Europe, North America, Philippines, Thailand, and Indonesia, have found ZEA in maize extraction [5]. Owing to its toxicity and potential risks for humans and animals, the European Union has set strict limitations on ZEA in food and feed. For example, the maximum limits are 100 µg kg^−1^ for unprocessed cereals and 350 µg kg^−1^ for unprocessed maize, respectively [6]. How to decrease production and adverse impacts of ZEA in maize represents a worldwide concern when food safety issues of consumers are considered.

For the detection of ZEA in foodstuff samples, a number of chromatographic methods are most used, including thin-layer chromatography (TLC) [7], high performance liquid chromatography (HPLC) [8], and liquid chromatography-mass spectrometry (LC-MS) [9]. These techniques, however, are usually high cost and time-consuming. In recent years, many immunoassay methods have been developed for rapid detection of ZEA, such as enzyme-linked immunosorbent assay (ELISA) [10], fluorescence polarization immunoassay [11], electrochemical microfluidic chips [12], immunochemical test [13], electrochemical magnetic bead-based inmunosensor [14], immunochromatographic strip [15], and so on. Among these immunoassay methods, ELISA has been extensively used as a cost- &time-saving, sensitive, quantitative, and high-throughput method. However, it is difficult to prepare ELISA samples, because the complex matrix of samples, especially with respect to agricultural products, could negatively affect the method accuracy.

ZEA is small molecule and is often found in low level. The complex matrix of maize could affect ZEA determination. Therefore, a highly-reliable and specific cleanup is required to simplify the sample preparation and improve the recovery effenciency. As a separation method, IAC can be conducted on a stationary phase that consist of an antibody coupled to a solid matrix, as well as antigen in a mobile phase. It provides a number of advantages over conventional extraction methods [16], such as the high specificity of the antibody for analyte, fast purification process, and effective reduction of toxic solvents, therefore, it performs well in the extraction of the target analyte.

In this study, IAC with antibody against ZEA was employed for sample preparation and IAC-icELISA was developed to detect ZEA contamination in maize. This method was based on the as-developed mAb 2D3 with highest sensitivity compared with previous literatures to our best knowledge. The developed IAC-icELISA method could be potentially extensively utilized in ZEA determination in agricultural products and food-stuffs.

## Materials and Methods

### Chemicals and instruments

ZEA, α-ZOL, β-ZOL, β-ZAL, Freund’s complete adjuvants (FCA), Freund’s incomplete adjuvants (FIA), hypoxanthine-thymidine (HT), polyethylene glycol 1450 (PEG1450, 50%), methyl cellulose, goat anti-mouse immunoglobulin horseradish peroxidase (IgG-HRP), 3,3′,5,5′-tetramethyl benzidine (TMB) and bovine serum albumin (BSA) were purchased from Sigma-Aldrich (St. Louis, MO, USA). The ZEA-BSA conjugates (2 mol ZEA per mol BSA) were purchased from aokin AG (Berlin, Germany). Roswell Park Memorial Institute (RPMI) 1640 medium with L-glutamine, penicillin, (+10,000 U mL^−1^) and streptomycin (+10,000 µg mL^−1^), HEPES (acid free, 238.3 g L^−1^), and Pierce® Rapid ELISA Mouse mAb Isotyping Kit were obtained from Thermo-Scientific (Rockford, USA). Fetal bovine serum (FBS) is from Sijiqing (Hangzhou, China). Water was obtained from a MilliQ purification system (Millipore), and all other inorganic chemicals and organic solvents were of the analytical reagent grade or above. The absorbance at 450 nm was detected using a SpectraMax® M2*^e^* Microplate Reader from Molecular Devices (Sunnyvale, USA). Agilent 1100 HPLC series consisted of a fluorescence detector and C_18_-column (3 µm particle size, 150 mm×2.1 mm I.D.).

This study was carried out in strict accordance with the recommendations in the Guide for the Care and Use of Laboratory Animals of the National Institutes of Health. The protocol was approved by the Laboratory Animal Monitoring Committee of Hubei Province and performed accordingly. All surgery was performed under sodium pentobarbital anesthesia, and all efforts were made to minimize suffering.

### Animals and cells

Female BALB/c mice were purchased from Centers for Disease Control and Prevention of Hubei Province (Wuhan, China). SP2/0 myeloma cells were purchased from China Center for Type Culture Collection (CCTCC, Wuhan, China).

### Breeding of hybridomas

Three six-week-old female BALB/c mice were immunized with ZEA-BSA conjugates. The immune and breeding procedure was described in our previous study [17]. After the fifth immunization, an intra-peritoneal booster without adjuvant was given to a mouse whose antiserum exhibited the highest titer and sensitivity three days before cell fusion. The spleen of the immunized female mice was aseptically removed and the freshly-isolated SP2/0-Ag14 myeloma cells were prepared. Spleen lymphocytes (10^8^) and SP2/0-Ag14 myeloma cells (10^7^) were fused in the presence of PEG1450. Two weeks after cell fusion, the white tops that could be seen by naked eyes were picked out of the monoclonal hybridomas and transferred to 96-well culture plates with the fresh complete medium containing HT. The anti-ZEA positive clones were checked in a two-step screening procedure [18] and ZEA was used as the competitive inhibitor.

### Producing and screening of antibodies

Ascitic fluid was produced by injecting hybridoma cells into the BALB/c mice, which had been given 0.4 mL FIA 7 days before. Then, the antibodies were purified by caprylic acid-ammonium sulfate precipitation as earlier described [19]. The isotypes of the monoclonal antibodies were classified using a commercially available kit from Sigma. The best working concentration of the anti-ZEA antibody was determined by indirect noncompetitive ELISA. Indirect competitive ELISA using ZEA as a competitor was used to search for IC_50_. The IC_50_ value was calculated to evaluate the sensitivity, which represented the concentration of the toxin that produced 50% inhibition of the antibody bound to the coating antigen [20]. The mAbs with higher titers and sensitivity were selected for expanding production and further assay.

### ELISA procedures

The icELISA method was used for analyzing ZEA. The coating antigen was diluted with 0.05 M carbonate-bicarbonate buffer (pH 9.6), and 100 µL/well of the antigen was added to the 96-well microtiter plate and incubated at 37°C for 2 h. After being washed with Phosphate Buffered Saline Tween-20 (PBST) three times, the plate was blocked with 1% ovalbumin (OVA) in PBST (200 µL/well) at 37°C for 1 h. After the plate was washed again, 50 µL/well of the analyte solution or samples and 50 µL/well of the mAb diluted with the PBST were added (100 µL/well) and incubated at 37°C for 1 h. Then, the plate was washed in the previous procedures, and the goat anti-mouse IgG-HRP (1∶5000 in PBST, 100 µL/well) was added and incubated at 37°C for 1 h. After being washed for six times, the plate was added with the TMB solution (9.5 mL of citric acid, 500 µL of 2 mg mL^−1^ TMB in ethanol (m/v), 32 µL of 3% H_2_O_2_) at 100 µL/well and incubated at 37°C for 15 min. The citric acid was 1.841 g Na_2_HPO_4_·12H_2_O, 0.933 g C_6_H_7_O_8_·H_2_O, and 100 mL triple distilled water. Then, the color developed was stopped by 2 M H_2_SO_4_ (50 µL/well) and the absorbance was measured at 450 nm.

### Optimization of icELISA

The optimum ZEA-BSA concentration required for coating onto the microtiter plate, and the best working concentration of the anti-ZEA antibody were determined using checkerboard titration. In the titration procedure, appropriate concentrations of the coating antigen were prepared with serial dilutions from 0.1 µg mL^−1^ to 1 µg mL^−1^ ZEA-BSA using a dilution factor of 2 in the carbonate-bicarbonate buffer. The monoclonal antibodies were serially diluted with 2-fold dilutions from 1∶5000 to 1∶640,000 in PBST. A set of experimental parameters including blocking reagents, pH, and ionic strength, were optimized sequentially to improve the sensitivity of the immunoassay as earlier reported [21]. Firstly, the influence of the blocking reagents (1% OVA, 1% BSA, 5% nonfat milk) was investigated. Then, the PBS solutions of different pH values ranging from 6.0 to 9.0 were evaluated. Finally, the PBS was tested with different salt concentrations to evaluate the effect of the ionic strength.

### Cross-reactivity to ZEA and its metabolites

To assess the cross-reactivity of the obtained mAb, the ZEA-related metabolites such as α-ZOL, β-ZOL and β-ZAL were used as competitive inhibitors in the icELISA method. The procedures were determined by dividing the 50% inhibitory concentration (IC_50_) values of ZEA by the IC_50_ values of each metabolite.

### Preparation and characterization of IAC

IAC was prepared according to the manufacturer's instructions and related literature [21]. A 10 mg mAb against ZEA was covalently coupled with 1 g CNBr-activated Sepharose 4B and packed into a cartridge (0.3 mL Sepharose per column). The capacity of the ZEA columns was determined by comparing the amounts of added ZEA with the IAC duplicate measurements detected using HPLC. The IAC stability was evaluated by spiking IAC with ZEA at concentrations of 10, 20, and 80 ng mL^−1^ in 20% methanol-water (v/v), and each concentration was prepared in three duplicates.

### Sample preparation

The blank maize sample was characterized using HPLC and then the spiking procedure was followed as previously reported [22] with only slight modifications. Then, standard ZEA solutions were added to the blank sample at concentrations of 5, 10, and 20 µg kg^−1^. The spiked samples were then finely ground using a laboratory mill, and 5 g of the samples was placed in a 50 mL centrifuge tube. 25 mL acetonitrile-water (90:10, v/v) containing 4% NaCl was added and blended at a high speed for 2 min. Then, the sample was extracted by ultrasonic at 50°C for 5 min before being filtered through two pieces of double filter paper. The filtrate of 2 mL was 1∶8 diluted with 14 mL distilled water and filtered through 0.45 µm filter membrane. 12 mL filtrate was collected in a glass tube, 10 mL of which was cleaned up and concentrated ten times using IAC at a flow rate of about 1 drop per second and the other 2 mL was analyzed using icELISA without the IAC cleanup step.

### Validation of IAC-icELISA

IAC was used against ZEA, which was eluted with 1 mL ethanol at the rate of 1 drop per second. Then, 100 µL elution was diluted and detected using icELISA with the optimized parameters. The other 900 µL elution was evaporated under a gentle nitrogen stream at 50°C until dry, and then it was suspended with 900 µL HPLC mobile phase and detected using HPLC. The HPLC system (Agilent 1100) was equipped with a C_18_-column (2.1 mm, 3 µm particle size) at the temperature of 35°C. The mobile phase consisted of acetonitrile and water at a volume ratio of 48∶52 and was eluted at the flow rate of 0.4 mL/min. The excitation and emission wavelengths of the fluorescence detector were set to 274 nm and 446 nm, respectively. The results from icELISA and IAC-icELISA were compared and studied to evaluate the matrix effect of the maize. The results from IAC-icELISA and HPLC were also compared for validation of this newly-developed method.

## Results, Discussion, and Conclusions

### Breeding of hybridomas

In the screening of the serum supernatants, all of the three mice showed high titers for ZEA after the fourth inoculation. With better sensitivity and titer, the third mouse was chosen for cell fusion. The colonies visible to the naked eye were transferred to individual wells in a 96-well culture plate containing the HT medium and cultivated at 37°C with 0.5% CO_2_. When the cells filled 2/3 of the wells, the supernatants were analyzed using indirect noncompetitive ELISA and icELISA for antibody secretion. Indirect noncompetitive ELISA indicated positive monoclonal hybridomas. Then, to select specific antibodies for ZEA, 25 ng mL^−1^ ZEA was used as competitors to screen the positive hybridomas to get ones with high-affinity to ZEA. Finally, five clones, named 2D3, 2D10, 3C3, 5F7, and 1D9, were screened and the results are listed in Table 1.

**Table 1:** Screening results of hybridomas.

		OD_450_ values^b^	
Clones	Titers of supernatants^a^	25 ng mL^−1^	0 ng mL^−1^
2D3	1500	0.08	0.876
2D10	1250	0.077	1.184
3C3	60	0.085	0.853
5F7	80	0.09	0.91
1D9	16	0.148	1.452

^a^ The titers of supernatants were defined as the reciprocal of the dilution that gave an absorbance closest to 1.0.

^b^ ZEA was prepared by diluting the standard solution with PBS, mixed with equivalent supernatants, the final concentrations were 25 ng mL^−1^, 0 ng mL^−1^ were PBS containing the same content of methanol.

### Characterization of antibodies

**Figure 1:**
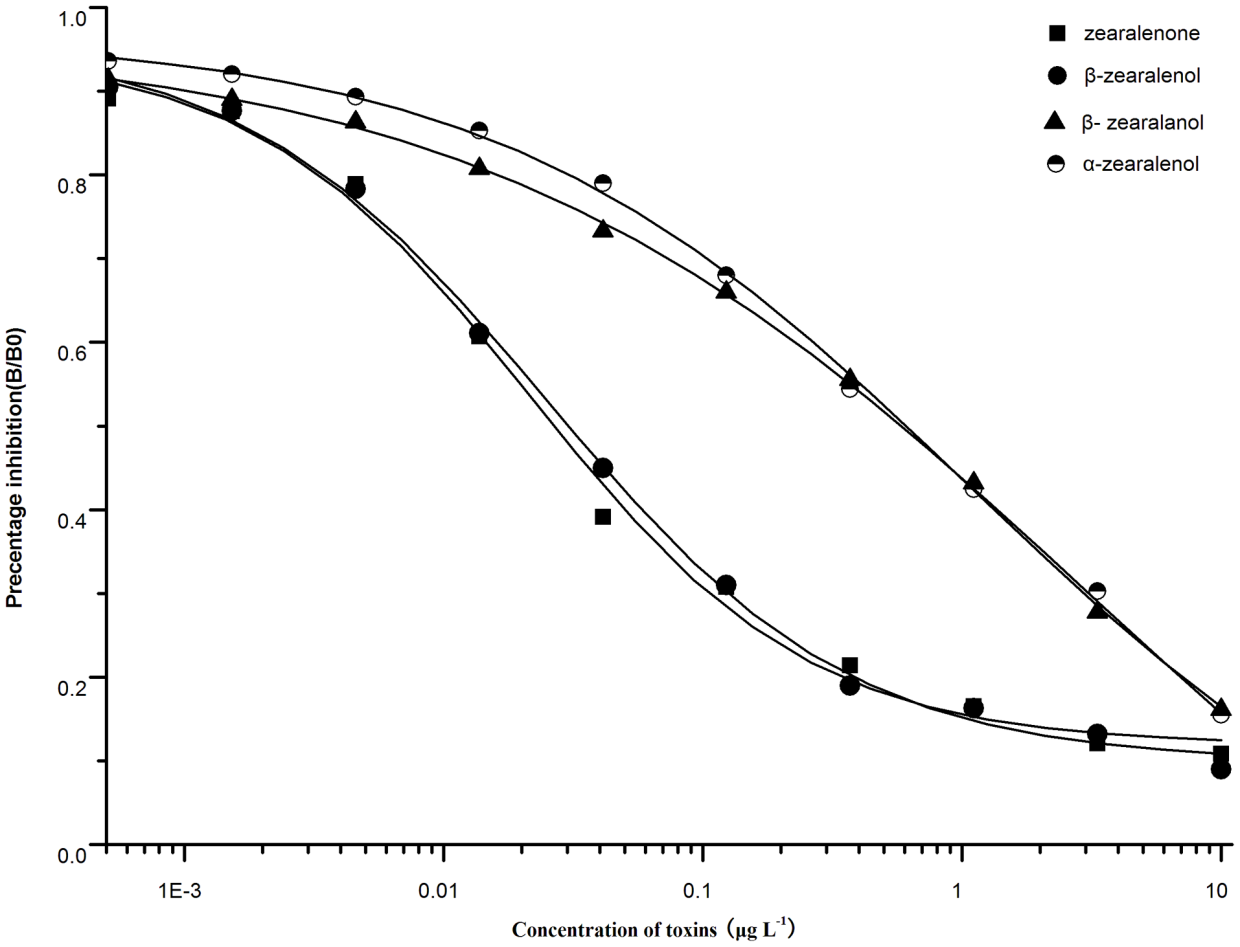
Cross-reactivity of 2D3 to ZEA, α-zearalenol, β-zearalenol, and β-zearalanol.

Five independent clones (2D3, 2D10, 3C3, 5F7, and 1D9), which could produce antibodies against ZEA, were injected into the BALB/c mice and then the ascetic fluid were purified and evaluated. The isotypes of the five mAbs were IgG. It is well known that sensitivity and specificity were the most important parameters for the antibodies and for their assay methods [23]. The IC_50_ value was the main criterion to evaluate the sensitivity of the mAbs, and the cross-reactivity revealed the specificity of the mAbs [24]. The titer results, IC_50_ results for each clone in Table 2 indicated that the titer of 2D3 (1.5×10^5^) was the highest among them. And the mAb 2D3 was of better sensitivity than others. Then, the mAb 2D3 was utilized for further experiment. The cross-reactivity of 2D3 against ZEA, β-ZOL, α-ZOL, and β-ZAL were 100%, 88.2%, 4.4%, and 4.6%, respectively (Fig. 1).

**Table 2:** Results of titers and sensitivity of the antibody assessment.

Clones	Isotype	Titers of the antibody^a^	IC_50_ value^b^(ng mL^−1^)	Negative control serum
2D3	IgG2b	1.5×10^5^	0.02	0.01
2D10	IgG2b	2.2×10^4^	0.24	0.02
3C3	IgG2a	2.4×10^4^	0.35	0.03
5F7	IgG2a	1500	1.04	0.02
1D9	IgG1	3800	3.13	0.03

^a^ The titers of the antibody were defined as the reciprocal of the dilution that gave an absorbance closest to 1.0.

^b^ Concentration at which binding of the antibody to the coating antigen is inhibited by 50%.

According to previous reports, many types of antibodities against ZEA were obtained. Compared with the reported mAb or polyclonal antibody (PcAb) in Table 3, the mAb developed in this work performed the lowest IC_50_ value and the lowest LOD value.

**Table 3:** Comparison of the most significant evaluation results in the best results of anti-ZEA that had already been published in recent years.

Literature no.	mAb/PcAb	IC_50_ value (ng mL^−1^)	LOD (ng mL^−1^)
			
-a	mAb	0.02	0.002
1 [25]	PcAb	5	1
2 [26]	PcAb	2	0.1
3 [27]	mAb	0.8	0.1
4 [28]	mAb	1.79	0.1
5 [29]	mAb	131.3	10

^a^ The data were from the clone of 2D3 obtained in this paper.

### Optimization of icELISA

**Figure 2:**
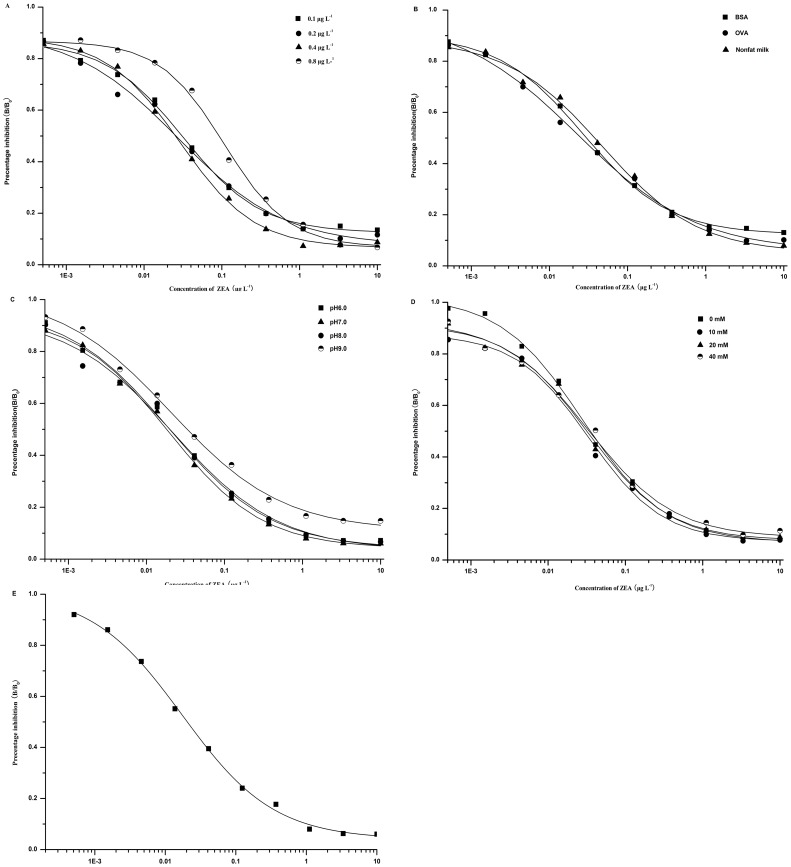
Influence of different factors. Including coating antigens and antibodies diluting ratio (A), blocking reagents (B), pH (C), and ionic strength (D) on the performances of the assay, standard curve for ZEA after optimization the factors (E), with the results being the means of three independent experiments

A satisfying compromise between the lowest detection capability and the minimum reagent expenses was obtained using 0.4 ng mL^−1^ ZEA-BSA and dilution of the antibody at the ratio of 1:152000 in the checkerboard titration procedure (Fig. 2A).

Three different physicochemical factors, including blocking reagents, pH, and ionic strength, were investigated. Blocking reagents have some effects on the sensitivity of the immunoassay (Fig. 2B). As can be seen from Fig. 2C, pH is one of the key factors that influence the assay characteristics and have effects on the icELISA procedure. Meanwhile, ionic strength has insignificant influences and ion concentration of 10 mM gives the best performance (Fig. 2D). Based on the favorable IC_50_ value, blocking reagents of 1% OVA, pH value of 7.0, and ion concentration of 10 mM were selected as the optimum for the assay. After optimization the factors the calibration curves was built (Fig. 2E). The IC_50_ value was 0.02 ng mL^−1^, the limit of detection (LOD), defined as the concentration corresponding to 85% of B/B_0_, was 0.002 ng mL^−1^.

### Characterization of IAC

The column capacity was determined by overloading the IAC columns with 10 mL standard ZEA solutions (20 ng mL^−1^) in methanol water (20:80) at the flow rate of 1 drop per second before being eluted with 1 mL ethanol. After that, the elution was analyzed using HPLC. The results indicated that the IAC capacity was 103 ng. The stability of the column was determined by carrying out recovery tests when ZEA at the concentrations of 10 µg kg^−1^, 20 µg kg^−1^, and 80 µg kg^−1^ was spiked with 20% methanol water (v/v). The assay was carried out in three duplicates. The recoveries were 93.4%, 99.1%, 99.6% respectively and with highly reproducible. Therefore, IAC could be used in developing immune methods to detect ZEA contamination.

### Matrix effect

In order to confirm the advantage of IAC in alleviating the matrix effect, the extracted compounds that were not cleaned up with IAC were analyzed using icELISA. And the sample extracts that were purified using IAC were analyzed with the icELISA method under the optimized conditions described above, as well as the HPLC method.

Recovery was expressed in terms of the percentage of the measured concentration compared with the fortified concentration. It was a function for the antibody specificity, concentration of the antibody on the column, and accessibility of the analyte to the antibody with the sample extracts passing through the column [18]. The ZEA recoveries obtained from icELISA without IAC, IAC-icELISA, and IAC-HPLC were summarized in Table 4. The recovery and repeatability results indicated that both IAC-icELISA and IAC-HPLC had high recovery rates and satisfactory reproducibility. However, the accuracy and repeatability of icELISA without IAC was much lower. The results indicated that the matrix could negatively affect analysis accuracy, but the use of IAC could efficiently alleviate the matrix effect and the developed IAC-icELISA method could be applied to ZEA detection in maize.

**Table 4:** Recovery analysis of ZEA in spiked maize by different methods.

	IAC-icELISA		Non-IAC-icELISA		IAC-HPLC	
Theoretical Level(µg kg^−1^)	ZEA±S.D.^a^	Recovery(%)	ZEA±S.D.^a^	Recovery(%)	ZEA±S.D.	Recovery(%)
5	4.50±0.03	93	2.30±0.01	46	5.44±0.13	108
10	9.10±0.02	87	5.40±0.02	54	9.83±0.27	98
20	16.6±0.01	83	10.4±0.04	52	18.70±0.53	94

^a^ Mean value±S.D. (n  =  3, S.D.  =  standard deviation).

## Conclusion

The new sensitive mAb against ZEA named 2D3 exhibited cross-reactivity of 100%, 88.2%, 4.4%, and 4.6% to ZEA, β-ZOL, α-ZOL, and β-ZAL, respectively. 2D3-based icELISA was performed in the 0.4 µg mL^−1^ ZEA-BSA, and the antibody (1000 ng mL^−1^) was diluted to 1:152000. With the optimized parameters, including blocking reagents, pH, and ionic strength of the icELISA, the IC_50_ value against ZEA was 0.02 ng mL^−1^, and the working range was 0.002–0.19 ng mL^−1^. Extraction of ZEA from maize using home-made IAC based on 2D3 has recoveries from 94% to 108% by HPLC detection. The IAC-icELISA method had a recovery of 83%–93%, whereas recovery of icELISA was as low as 46%–54% without sample cleaned up using IAC. The results indicated that IAC performed well in the sample cleanup and could improve the accuracy of assay. Therefore, the produced mAb 2D3 and developed IAC-icELISA method are effective in accurately determining ZEA in maize.
